# GNSS evaluation of GRACE-assimilated water storage models over 89 river basins worldwide

**DOI:** 10.1038/s41598-025-31887-1

**Published:** 2026-01-29

**Authors:** Majid Abbaszadeh, Tonie van Dam

**Affiliations:** 1 Faculty of Science, Technology and Medicine, University of Luxembourg, Luxembourg, Luxembourg; 2 College of Science, Geology and Geophysics, University of Utah, Salt Lake City, USA

**Keywords:** Hydrological loading, GNSS, GRACE, Data assimilation, Climate sciences, Environmental sciences, Hydrology

## Abstract

The gravity recovery and climate experiment (GRACE) and GRACE follow-on (GFO) gravity observations have significantly improved our understanding of the terrestrial water cycle. However, GRACE-assimilated (GA) hydrological models still differ significantly. This paper uses global navigation satellite system (GNSS) data to assess two global GA datasets: Global land water storage release 2 (GLWS2.0) and catchment land surface model GRACE data assimilation (CLSM-DA). From 2004 to 2019, the mean annual amplitude of equivalent water thickness (EWT) of these datasets differs by more than 25 mm over 40% of the modeled land area, and the timing of peak water storage diverges by as much as 30-days across 50% of their domain. We compare the modeled hydrological loading vertical displacement predicted from these models with GNSS uplift data to compare and contrast the model quality. Using river basin boundary information from 89 rivers, we cluster 9,163 global GNSS stations, each with at least three years of daily data. Results show that CLSM-DA generally agrees better with GNSS data across more river basins. Its 100–300 mm larger annual water variation accounts for better agreement in Africa, Southeast Asia, and parts of South America. In regions like the Western United States and Eastern Europe, where both models estimate similar annual amplitudes, CLSM-DA’s 30–60 day phase delay improves alignment with GNSS. This evaluation also reveals key limitations in both models, especially during extreme hydrological events such as droughts, and highlights the value of geodetic observations in advancing GA hydrological modeling.

**Supplementary Information:**

The online version contains supplementary material available at 10.1038/s41598-025-31887-1.

## Introduction

Hydrological models have been developed to describe terrestrial water storage (TWS) and its temporal variations. These models solve the water balance equations for various TWS components, incorporating climate information, land cover, and topography data. While hydrological models rely on physical assumptions about hydrological systems, GRACE provides direct but regional-scale (~ 350 km), monthly observations of TWS mass changes^1–3^. Though coarse, GRACE/GFO data offer valuable long-wavelength constraints for finer-scale models via data assimilation^4–7^. However, GRACE-assimilated (GA) products vary across centers due to model imperfections as well as data weighting and downscaling scenarios in assimilation implementation^8^.

To assess the quality of GA TWS anomaly (TWSA) data sets, in situ groundwater storage anomalies, river discharge, and soil moisture are typically used^4,9^. While these data serve as valuable benchmarks, they are poorly observed in many regions. Hence, the quality assessment of GA hydrological models, especially at the regional and global scales, is an ongoing research challenge.

We use GNSS data to assess GA models because: (1) GNSS reliably captures the Earth’s elastic response to regional TWS changes^10–12^, and (2) dense, long-term GNSS networks exist across many major river basins^13^. These features enable inversion of GNSS data to infer regional TWS variations. For example^14^, used GNSS displacements to localize water mass changes, while^15^ identified 0.1–0.6 m EWT variation in California. ^16^ linked GNSS-measured uplift to ~ 240 Gt of water loss in the western U.S, and ^17,18^ further highlighted topography-correlated TWS changes and deep moisture loss. More recently,^19^ used GNSS to model regional unloading during the 2012–2016 Great Salt Lake drought.

We compare and contrast two GA hydrological datasets: GLWS2.0 and CLSM-DA, developed by^20,21^, respectively. This study builds on the work of^20^, but with some differences: (1) we compare both models (not just GLWS2.0) with GNSS data; (2) we cluster GNSS stations based on the river basin boundary to evaluate the regional agreement between the GA models and GNSS data; (3) unlike^20^, who used GNSS data collected only at International GNSS Service (IGS) stations, we use the Nevada Geodetic Laboratory (NGL) dataset which includes data from about 20 times more stations, thereby facilitating a more detailed regional model assessment.

This analysis has two main objectives: (1) to highlight the GLWS2.0 and CLSM-DA inter-model inconsistency across large-scale river basins, and (2) to assess their respective performance in reproducing GNSS-measured hydrological loading displacements at the river-basin scales. To achieve these goals, we describe the TWS variations provided by the two models and their expected load-induced surface displacements in Sect. 2. This section also reviews surface displacement measurements using GNSS and evaluates the consistency of the GNSS dataset with the most recent reanalysis data product of the IGS. Section 3 explains the GNSS station classification and presents the numerical results on the agreement between modeled displacement and GNSS data. Section 4 discusses the results and concludes the paper.

## Terrestrial water and its induced displacement

### GLWS2.0 and CLSM-DA datasets

GLWS2.0 provides a monthly global 0.5°×0.5° dataset of TWSA from 2003 to 2019. GLWS2.0 is computed by assimilating GRACE-derived TWSA data into predictions made by ‘WGHM2.2e’^22^, the latest version of WaterGAP global hydrological model (WGHM)^23,24^. The TWSA data used in GLWS2.0, are calculated with respect to the TWS temporal mean from 2003 to 2016. The GSWP3-W5E5 daily precipitation, temperature, and solar radiation input data^25^ are used in WGHM2.2e. The water storage outcome of WGHM2.2e is calibrated against mean annual streamflow data from 1,509 sites. GLWS2.0 uses the GRACE TWSA data that is computed from level 2 spherical harmonic geopotential coefficients at the Technical University of GRAZ^26,27^.

CLSM-DA is a global daily 0.25°×0.25° model that includes TWS data from February 1, 2003, to the present. CLSM-DA is computed by assimilating GRACE TWSA data into the Catchment Land Surface Model (CLSM)^28^ version Fortuna 2.5 (CLSM-F2.5) which uses ECMWF climate input data. The GRACE TWSA data used in CLSM-DA is derived from the regional mass concentration (mascon) functions at the Center for Space Research (CSR) at the University of Texas^29^. In this study, we average the daily CLSM-DA grids into monthly averages of the CLSM-DA to maintain a consistent temporal resolution with GLWS2.0, and then use the two datasets at their native spatial resolution for the displacement forward modeling.

### Model consistency evaluation

#### Terrestrial water storage

Since the long-term linear trend of the GNSS vertical displacement time series is influenced by tectonic motion and glacial isostatic adjustment (GIA), we remove the long-term trend from the GNSS data. However, this also cancels the long-term linear variation of hydrological loading driven by climate warming in the GNSS measurements. To maintain consistency with the GNSS data, we similarly remove the mean and linear trend from GA TWS data for the period from January 1, 2004, to December 31, 2019, where the two datasets overlap.

We use the difference in seasonal TWSA between GLWS2.0 and CLSM-DA, along with predicted load-induced displacement (explained in the next subsection), to illustrate the inter-model inconsistencies. Seasonal signals are extracted via least squares fitting of a sinusoid with annual and semi-annual periods, allowing for constant but unknown amplitude and phase, at each grid point. To align spatial resolutions, GLWS2.0 results (0.50°×0.50°) are interpolated to 0.25°×0.25°, matching CLSM-DA.

**Fig. 1:**
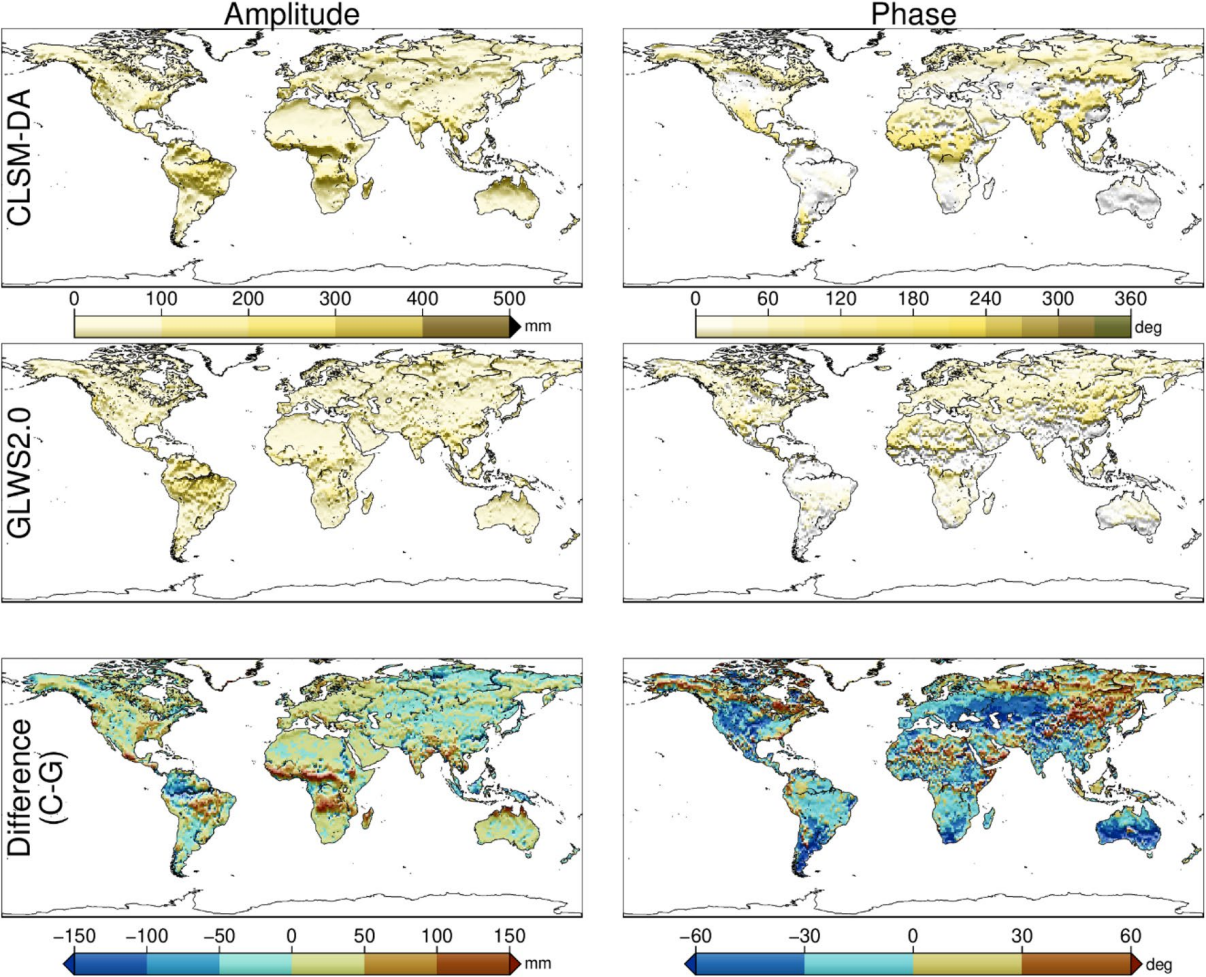
Annual amplitude and phase of a fitted sinusoid (comprising annual and semi-annual components) to predicted equivalent water thickness at each 0.25° × 0.25° hydrological cell from 2004.0 to 2020.0. The estimated amplitude and phase from each dataset are shown in the top and middle rows, labeled by the corresponding model name (CLSM-DA and GLWS2.0). The bottom panels display the spatial differences in amplitude and phase between the models (CLSM-DA minus GLWS2.0). This map is generated by Generic Mapping Tools (GMT) version 6.6.0^35^ available at https://docs.generic-mapping-tools.org/latest/.

Figure 1 shows the predicted annual TWSA amplitude in equivalent water thickness (EWT). Both models display broadly similar spatial patterns, though CLSM-DA exhibits smoother variability, likely reflecting greater GRACE data influence due to higher assimilation weights (Jürgen Kusche, personal communication). Notable inter-model amplitude differences (up to 100 mm) appear over the Southern Amazon, Niger, Congo, and Ganges basins. Phase differences of 30°–60° (~ 30–60 days) are also evident in Eastern Europe and the Western U.S.

#### Predicted loading displacement

The redistribution of terrestrial water mass imposes time-variable surface pressures on the Earth, causing the Earth’s surface to displace elastically. Given a model for the temporal variability of continental water mass, the theory of Green’s function, as described in^30^, can be applied to model the hydrological load-induced displacement^10,31,32^. We adopt the Preliminary Reference Earth Model (PREM) Green’s function computed by^33^ to model the vertical displacement in the center of the figure (CF) fame, consistent with the GNSS data coordinate frame^34^. The modeled displacement is based on TWSA gridded data that are corrected for their mean and linear trend at each grid cell.

**Fig. 2:**
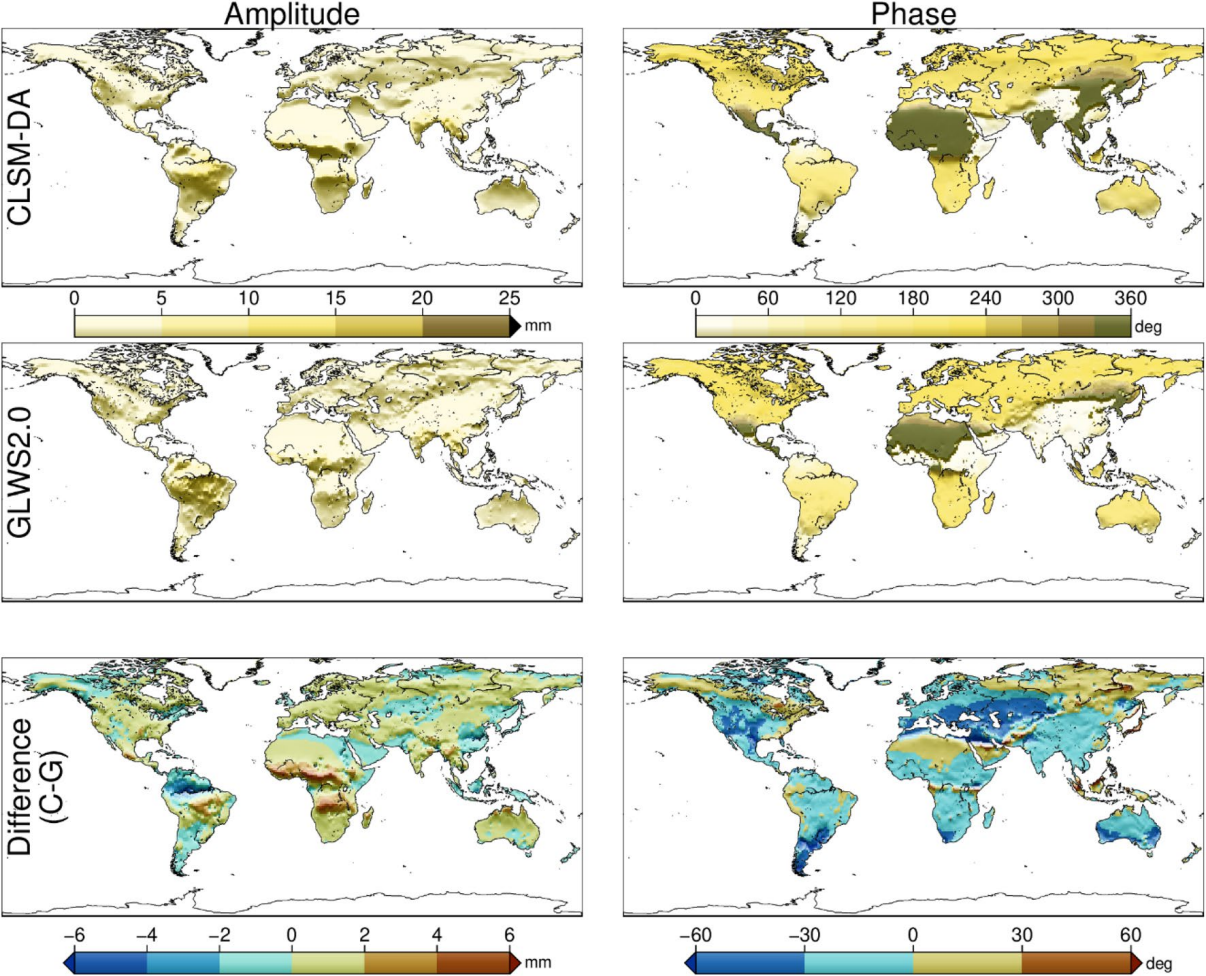
Same as Fig. 1, but for the predicted vertical elastic loading displacement derived from the CLSM-DA and GLWS2.0 datasets and the PREM Earth model. This map is generated by Generic Mapping Tools (GMT) version 6.6.0^35^ available at https://docs.generic-mapping-tools.org/latest/.

To compare load-induced displacements computed by the GA models, we compute surface displacements on a global 0.25°×0.25° grid. As in previous subsection, we fit a combined annual and semi-annual sinusoid to the displacement time series at each cell. Figure 2 shows the resulting annual amplitudes and phase angles. It can be seen from the figure that the spatial patterns in displacement amplitude and phase closely resemble the corresponding TWSA variations in Fig. 1.

The bottom panels of Fig. 2 show that inter-model differences in water variation propagate into both displacement amplitude and phase. For example, CLSM-DA predicts seasonal displacement amplitudes ~ 5 mm larger than GLWS2.0 in the Niger and Congo basins. This larger than 10 mm peak-to-peak differences exceed typical GNSS noise levels, highlighting GNSS ability to help assess model accuracy. The observed phase shifts of the displacement field are consistent with those in TWSA (Fig. 1, bottom panel).

### GNSS-derived hydrological loading displacement

#### GNSS data processing overview

We use the GNSS-measured ground station height time series in the IGS14 coordinate frame, provided by the Nevada Geodetic Laboratory (NGL) at the University of Nevada, Reno^13^. NGL uses GipsyX version 1.0^36^ to perform precise point positioning (PPP)^37^ using undifferenced ionospheric-free data. In the NGL’s PPP processing, satellite clock, orbit, and attitude, as well as Earth orientation parameters, are fixed to the values provided by the Jet Propulsion Laboratory (JPL) analysis center. Phase center variations of the satellite transmitters and ground receiver antennas are corrected using IGS ANTEX files (IGSMAIL-7399), and a priori zenith tropospheric delay is modeled using Vienna Mapping Function Version 1 (VMF1)^38^, whilst the residual tropospheric delay is estimated in the PPP processing. The NGL’s PPP solutions are corrected for solid Earth tide, ocean tide, and pole tide according to the IERS2010 conventions^39^, although they do not account for loadings induced by non-tidal atmospheric and ocean bottom pressure, as well as surface hydrology.

#### GNSS observation of hydrological loading

To derive hydrological loading effect from GNSS displacement data, we remove non-tidal atmospheric loading (NTAL) and non-tidal ocean loading (NTOL) effects, by applying corrections computed by the Earth System Modeling Group of GeoForschungsZentrum (GFZ) Potsdam (ESMGFZ)^40^. The ESMGFZ atmospheric loading correction has the advantage of using ECMWF climate data, which is also applied for the tropospheric delay correction in the NGL PPP processing^41^.

We also remove the linear trend from the GNSS time series to account for tectonic and GIA effects. The linear trends are estimated using the Median Interannual Difference Adjusted for Skewness (MIDAS) algorithm^42^. Some of the GNSS time series may also be contaminated by abrupt level change, likely due to earthquakes or instrument/processing changes. Hence, we applied offset correction in two steps: (a) extraction of time of potential offsets from the station step file, available on the NGL website (geodesy.unr.edu/NGLStationPages/steps.txt, Date of access: 27 January 2025), and (b) fitting a combined annual and semi-annual sinusoidal function with a collection of Heaviside steps at the times of documented steps, as shown in Eq. (1):

1 $$\:y={A}_{1}\mathrm{sin}\left(2\pi\:t+{\phi\:}_{1}\right)+{A}_{2}\mathrm{sin}\left(4\pi\:t+{\phi\:}_{2}\right)+\sum\:_{i=1}^{{N}_{step}}{B}_{i}.H(t-{t}_{step,i})\:\:\:\:\:\left(1\right)$$

In this equation, the first two terms include annual and semi-annual sinusoids and the last term accounts for the time series level change at documented step epochs. Amplitude and phase of the sinusoids [$\:{A}_{1},\:{A}_{2},\:{\phi\:}_{1},\:{\phi\:}_{2}]$ and the magnitude of the level change at the i-th reported offset ($\:{B}_{i})$ are unknown and need to be estimated.

Most of the detrended GNSS displacement time series contain a strong annual signal presumably caused by the annual redistribution of terrestrial water, but our model of Eq. 1 fails to capture effects such as post-seismic displacement observed at some sites. Furthermore, there are stations with unusually large number of reported steps, mainly due to the NGL’s experimental formula of using distance from GNSS stations to earthquake epicenters to alarm steps. For these stations, it is not unlikely to have steps concentration in a segment of the time series, making the model of Eq. 1 ill-posed.

Hence, we use the condition number from the regression step^43^ to identify and exclude stations with poor offset parametrization. As shown in Figure A1 of the supplementary material, stations with a condition number greater than 10¹⁵ displays a rapid increase in the RMS of the data after offset correction, implying a non-realistic offset estimation. Therefore, we adopt 10¹⁵ as a conservative threshold to exclude ~ 3% of stations that cannot be robustly modeled by Eq. (1).

Finally, we compute the monthly means of the detrended and offset-corrected GNSS data which is comparable to the temporal sampling of the GA predictions.

#### Quality assessment of GNSS data

**Fig. 3:**
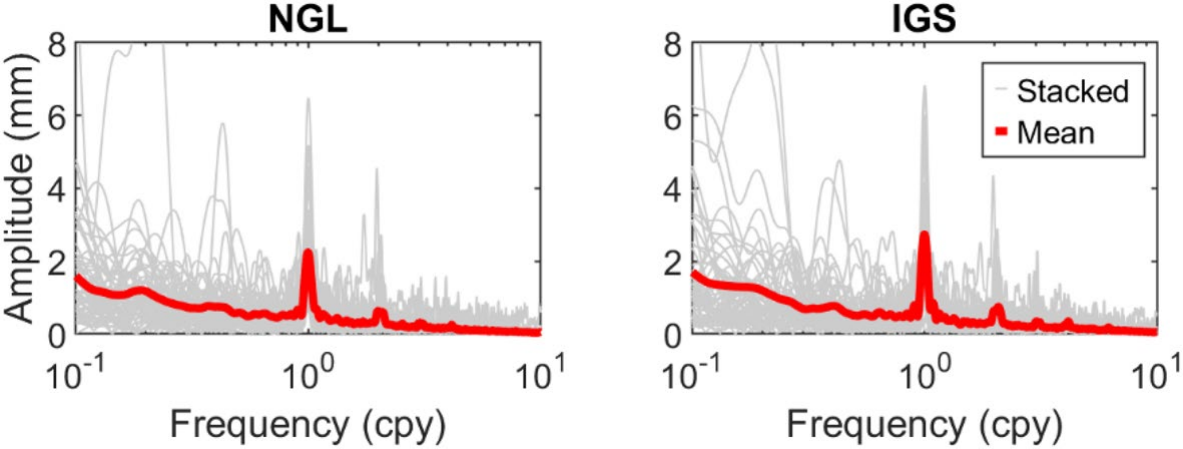
Stacked (gray) and mean (red) amplitude spectrum of the vertical displacement time series for IGS core stations computed by NGL and IGS repro3 solutions. The time series are corrected for NTAL and NTOL, jumps, mean, and linear trend and they are filtered for the sub-monthly variation.

To ensure the quality of the NGL time series, we examine it relative to the product generated from the IGS repro3 combined solution^44^. To this end, we compute vertical displacement from IGS repro3 SINEX files and then treat the time series for the non-hydrological loading effects and linear trend, as we do for the NGL data. We then generate the amplitude spectrum of the vertical displacement time series from the two solutions. The results at those IGS stations that have been used for the ITRF2014-ITRF2020 transformation (itrf.ign.fr/docs/solutions/itrf2020/core-network_ITRF2020.txt, Date of access: 27 January 2025) and contain at least 10 years of data are shown in Fig. 3.

It can be seen from the figure that the NGL time series are slightly noisier over the seasonal and sub-seasonal frequency bands. However, this does not bias the model performance evaluation, as shown in Figure A2 of the supplementary material. Figure 3 also depicts large amplitude of the low-frequency spectra, i.e., frequency between $\:{10}^{-1}\:\mathrm{t}\mathrm{o}\:{10}^{0}\:\mathrm{c}\mathrm{y}\mathrm{c}\mathrm{l}\mathrm{e}\:\mathrm{p}\mathrm{e}\mathrm{r}\:\mathrm{y}\mathrm{e}\mathrm{a}\mathrm{r}\:\left(\mathrm{c}\mathrm{p}\mathrm{y}\right),$ in both solutions, which are likely due to the unresolved offsets^44,45^.

#### GNSS station selection for model assessment

To identify the most appropriate stations from both datasets for model assessment, we first remove stations located in islands with area smaller than the GLWS2.0 cell size. We use Global Self-consistent Hierarchical High-resolution Shorelines version 2.3.7 (GSHHS2.3.7) data^46^ for the island information. Thereafter, stations with unsuccessful offset correction, i.e. those with a condition number of the offset estimation procedure greater than $\:{10}^{15}$, are discarded. We also check the completeness of the GNSS time series within each month, consistent with the GLWS2.0 temporal resolution. Monthly data that contains at least 80% of the expected GNSS daily solutions are counted. Eventually, we exclude a station if it contains fewer than 36 monthly data arcs or if there is a negative correlation between the modeled and GNSS-derived displacement. A negative correlation can indicate that the observed displacement is anti-phase with the elastic loading prediction, a signature frequently attributed to poroelastic processes. We also discard stations in Greenland where the two models have no reliable information on terrestrial water variation. The GNSS data check is performed from 2004.0 to 2020.0 to match the time frame of the GA models.

Out of 20,644 and 1,156 stations processed by NGL and IGS, respectively, 9,163 and 851 stations satisfy the above-mentioned criteria. Hence, we use the NGL data product for the rest of this paper.

## Assessment of GLWS2.0 and CLSM-DA against GNSS data

### Model assessment metrics

For GNSS stations situated in regions where non-hydrological and poroelastic deformation is negligible or well-constrained, the vertical land motion observed in GNSS time series can be substantially attributed to surface mass loading, primarily from hydrological sources. Therefore, removing modeled hydrological loading displacement from the GNSS observations should reduce the variance of the residual signal if the model accurately represents the true loading. This principle underpins the use of root mean square (RMS) reduction of the GNSS-load-corrected displacement time series as a diagnostic metric to assess the consistency between modeled loading and observed displacements^10,47^.

In this study, we use the TWSA outputs from the two GA models to compute the corresponding vertical elastic loading displacements at GNSS stations. These modeled displacements are then subtracted from the GNSS-observed vertical displacement time series, and the performance of each dataset is quantified by the percentage reduction in RMS (RMSr), defined as:

2 $$\:RMSr=\frac{{RMS}_{G}-{RMS}_{G\mathrm{*}}}{{RMS}_{G}}\times\:100$$

where $\:{RMS}_{G}$ and $\:{RMS}_{G\mathrm{*}}$ represent the RMS of the original and corrected GNSS time series, respectively.

While RMSr provides a useful indication of how much variance in GNSS displacement is explained by the modeled loading, it can also be influenced by amplitude mismatches between model and observation. For instance, if the model and GNSS time series are perfectly in phase but the model amplitude is slightly overestimated, loading correction can yield a negative RMSr despite strong temporal coherence. Therefore, RMSr should be interpreted together with the correlation coefficient, which emphasizes phase and shape agreement independently of amplitude differences.

### Overall model assessment

**Fig. 4:**
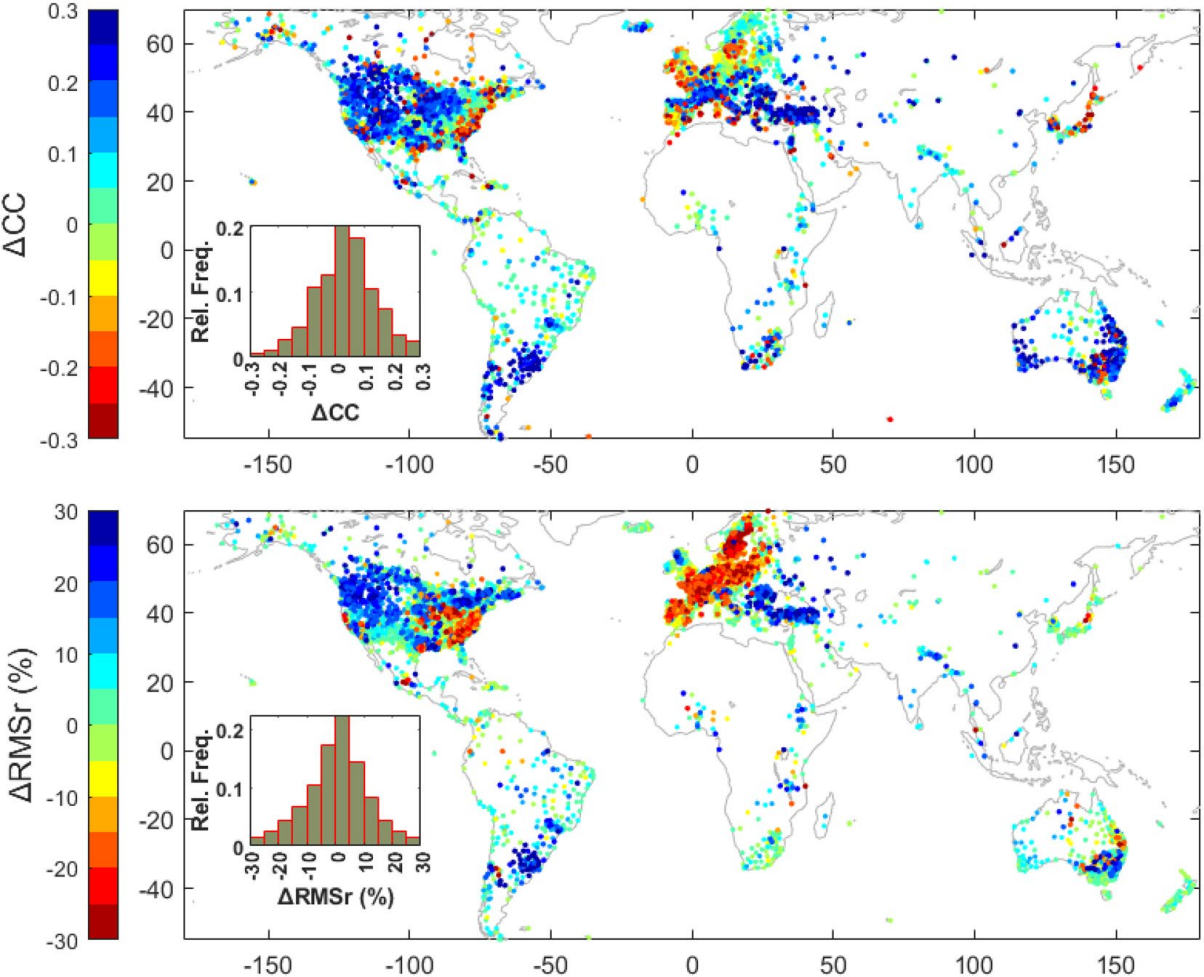
Relative agreement of model-derived hydrological loading with GNSS vertical displacements. Top) Difference in correlation coefficients between GNSS and CLSM-DA versus GNSS and GLWS2, Bottom) Difference in the percentage RMS reduction of GNSS data after removing loading effects computed by CLSM-DA and GLWS2. Positive (blue) values indicate better agreement of CLSM-DA with GNSS, whereas negative (red) values indicate better agreement of GLWS2 with GNSS. This map is generated by MATLAB (R2022b)^48^.

To evaluate the relative performance of the two models, we subtract the percentage RMSr of the GNSS data corrected using GLWS2.0 from the RMSr resulted by CLSM-DA. We also subtract the correlation coefficient between GNSS and GLWS2.0 loading displacement from that between GNSS and CLSM-DA, and the results are shown in Fig. 4.

Figure 4 illustrates that the model-GNSS agreement difference based on the two metrics yield an overall consistent result. However, the stronger correlation of CLSM-DA to GNSS in Central Europe is not equivalent to a greater RMSr, likely due to a non-realistic amplitude of the modeled water variations. The figure also indicates that the RMSr and correlation difference caused by the two models differ regionally up to 30% in several parts of the globe, as shown by dark red or blue. This motivates a regional assessment of the models, within spatial framework of river basins. Indeed, river basins are natural units of water storage and flow data which are used in the model computation and calibration^22^.

### Regional model assessment

#### GNSS stations clustering

We use Level 3 sub-basins from the HydroBASINS dataset^49^ to cluster GNSS stations, regarding GRACE data product resolution and GNSS station density. Sub-basins with ≥ 10 stations are selected in North America and Europe, and ≥ 5 elsewhere. To assess basin-scale water storage, we compute the common mode component (CMC) of GNSS-derived and modeled hydrological loading displacements^50^. The difference between GNSS-derived and modeled CMC is a representative of the regional model-GNSS misfit. The CMC is estimated using the stacking method of^51^, with the network median used to reduce sensitivity to unresolved offsets in GNSS data. We analyze data from 2010 to 2019, when the coverage of GNSS network peaked in most basins.

**Fig. 5:**
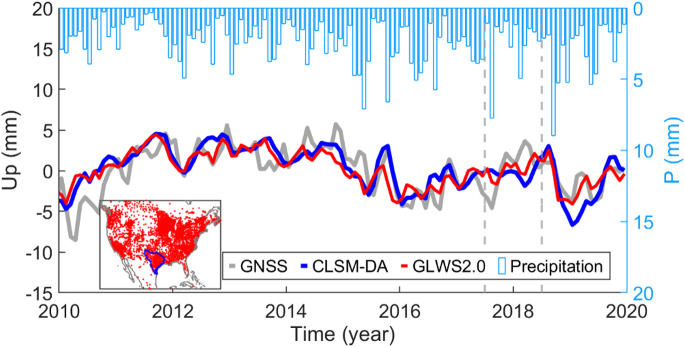
Common mode component (CMC) of the displacement, derived from GNSS data and predicted by CLSM-DA and GLWS2.0 models, along with regional monthly average precipitation at GNSS stations in Gulf of Mexico Seaboard watershed (blue polygon in the map). The GRACE data gap period is marked by vertical lines.

An example plot for the displacement CMC and regional precipitation in the Gulf of Mexico Seaboard is shown in Fig. 5. Precipitation data are obtained from the ERA5 reanalysis dataset, which provides hourly gridded precipitation fields from the ECMWF’s fifth-generation global climate reanalysis^52^. The data are first downsampled to monthly means, then interpolated spatially to the locations of GNSS stations for each month. Finally, a regional monthly median is computed across all GNSS stations to represent the spatially aggregated precipitation signal.

Figure 5 confirms a general agreement between GNSS-observed and modeled loading displacement at regional scale. It also indicates that during GRACE data gap period (shown by vertical lines), the two models show a greater divergence from GNSS observations. Furthermore, the GNSS-derived CMC shows stronger sub-seasonal variations, possibly due to local water variations that are not captured by the GA models.

Figure 5 also displays an anti-correlation between precipitation and land uplift. For instance, GNSS data shows about 15 mm of regional land uplifts from April 2010 to December 2011, due to terrestrial water loss during the first year of the 2010–2014 drought in Texas. The figure also demonstrates that loading induced by heavy regional rainfalls, such as those that occurred in May and October-November 2015 are followed by a lagged land subsidence.

#### Regional agreement between models and GNSS data

For the rest of this section, we focus on the regional agreement between models and GNSS data in selected river basins. Further to the RMSr of the GNSS-load-corrected data, we look at the correlation coefficient between sub-seasonal GNSS-derived and modeled displacement.

To extract the sub-seasonal land oscillation, we use wavelet analysis, as suggested by^20^. This process involves performing a wavelet transformation on the CMC time series, followed by an inverse transformation on the spectra with periods between 90 and 150 days. We use MATLAB’s cwt and icwt functions with a Morlet (Gabor) kernel for the analysis^48^. To mitigate edge error, we remove the first and last 150 points from the reconstructed sub-seasonal displacement time series. The results for the correlation coefficient between modeled and GNSS-derived sub-seasonal loading displacement (per river basin) are shown in Figure A3 of the supplementary material.

##### North America

**Fig. 6:**
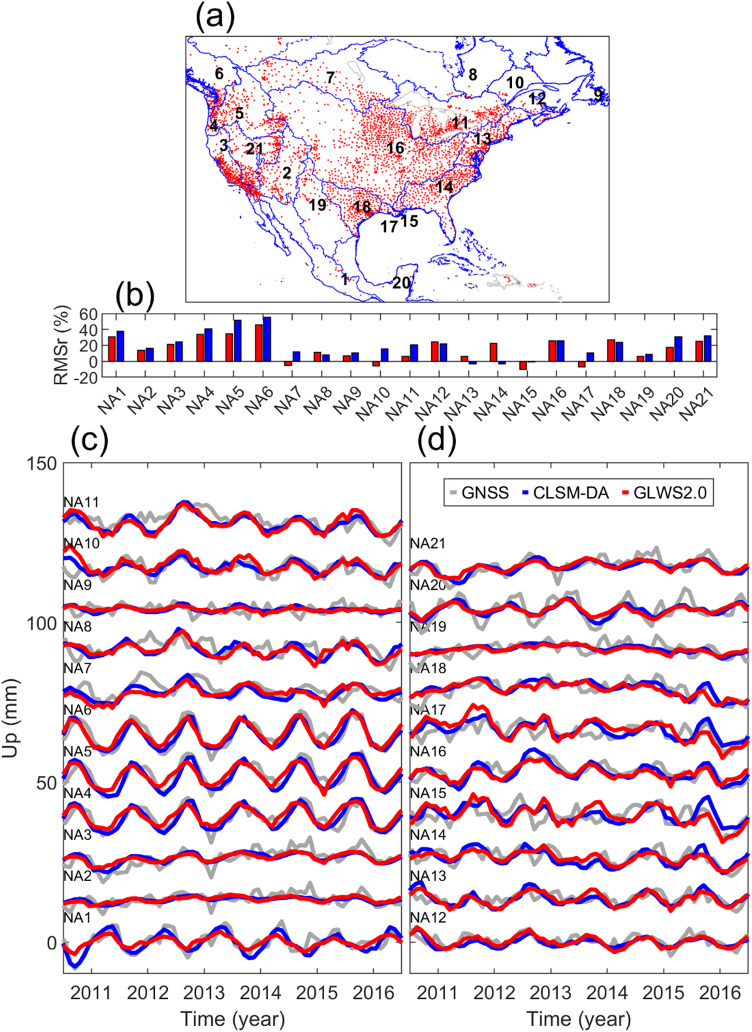
(**a**) Boundary of river basins in North America (derived from Level 3 HydroBASINS data product) with GNSS stations inside the basins, (**b**) RMS reduction of the GNSS-derived common mode component (CMC) after removing the modeled CMC (**c**) CMC of the GNSS-observed (gray) and modeled hydrological loading vertical displacement from CLSM-DA (blue) and GLWS2.0 (red), (**d**) same as panel (**c**), but for additional basins.

The boundaries of river basins and filtered GNSS stations in North America are illustrated in Fig. 6a. In this figure, the basins which are labeled by a number, are considered for model assessment. Figure 6b compares the RMSr of the GNSS-derived displacement CMC after removing that computed by GA models. The time series of the GNSS-measured and modeled CMC are also shown in Fig. 6c and d. Although the displacement time series from 2010.5 to 2016.5 is plotted, all available data from 2010 to 2019 are used for model assessment. We plot a shifted time series for clarity, and we also use the plotting convention in Fig. 6 for other continents.

Figure 6b depicts that the RMSr of the GNSS-loading-corrected data varies across models and basins. We attempt to explain this variation with the help of the displacement time series. We start from NA5 (Columbia River basin) where the modelled displacement shows one of the greatest agreement with GNSS data. Figure 6c shows strong seasonal displacement in NA5, which is confirmed by the region’s strong seasonal precipitation. However, the maximum annual subsidence predicted by CLSM-DA occurs about 30 days after that proposed by GLWS2.0. In this basin, the loading correction computed by CLSM-DA reduces the RMS of GNSS data by ~ 55% which is ~ 20% larger than the result of GLWS2.0. We remove the phase lead of GLWS2.0 loading displacement and then correct GNSS observation with it. The test identifies ~ 10–20% improvement in the RMSr of the GNSS data across NA5 and its neighbors i.e., NA4, NA6, NA21, and NA7. This implies that CLSM-DA models the time of water variation more accurate than GLWS2.0 in Western North America. Our finding is consistent to that reported by^53^ about an average 38 days delay of the maximum GNSS-inverted TWSA relative to the maximum snow water equivalent in Western United States.

In many basins in North America, extending from southwest to northeast of the continent, the two models have negligible difference in recovering GNSS data. However, GLWS2.0 better reduces the RMS of GNSS data in NA12, NA13 and NA14, located in Atlantic Ocean and Gulf of Mexico Seaboard. We suspect that some part of this better agreement is from the sub-seasonal water loading, as we found larger correlation between sub-seasonal variations of GLWS2.0 and GNSS data in NA13 and NA14.

In few river basins in North America, the GNSS-measured hydrological loading displacement deviates significantly from its modeled values. For instance, Fig. 6c shows that the GNSS-derived and predicted seasonal uplifts and subsidence in NA3 are in phase, but GNSS data shows more extreme variation. This is also seen in the neighbor river basins (i.e., NA2, NA18, NA19, and NA21). We inspected the entire displacement time series from 2004 to 2019 (which is not shown in Fig. 6) and found that the model-GNSS deviation becomes smaller outside the 2011–2016 drought period in the Western United States. Similar underestimations of water storage variations by about 30–50% have been reported for this region^15,17,18^, mainly due to the underestimation of snow components in global hydrological models. This deficiency likely becomes more pronounced during drought periods, when snowpack anomalies dominate the regional water storage variability.

Figure 6d shows that the GNSS-derived and modeled displacement difference is also large in NA15 and NA17, as the two smallest level 3 basins in North America. We found that only two or three GNSS stations were operational in these basins before 2014, with some unresolved data offsets in NA15. Although the model-GNSS agreement has improved from 2014 onwards in those basins, it is not as good as their neighbors, i.e., NA14 and NA16. We think that the GNSS-derived loading displacement CMC in NA15 and NA17 is mainly affected by local water variation. This is supported by one of the largest correlation between modeled and GNSS-observed sub-seasonal displacement in NA17.

##### Europe

**Fig. 7:**
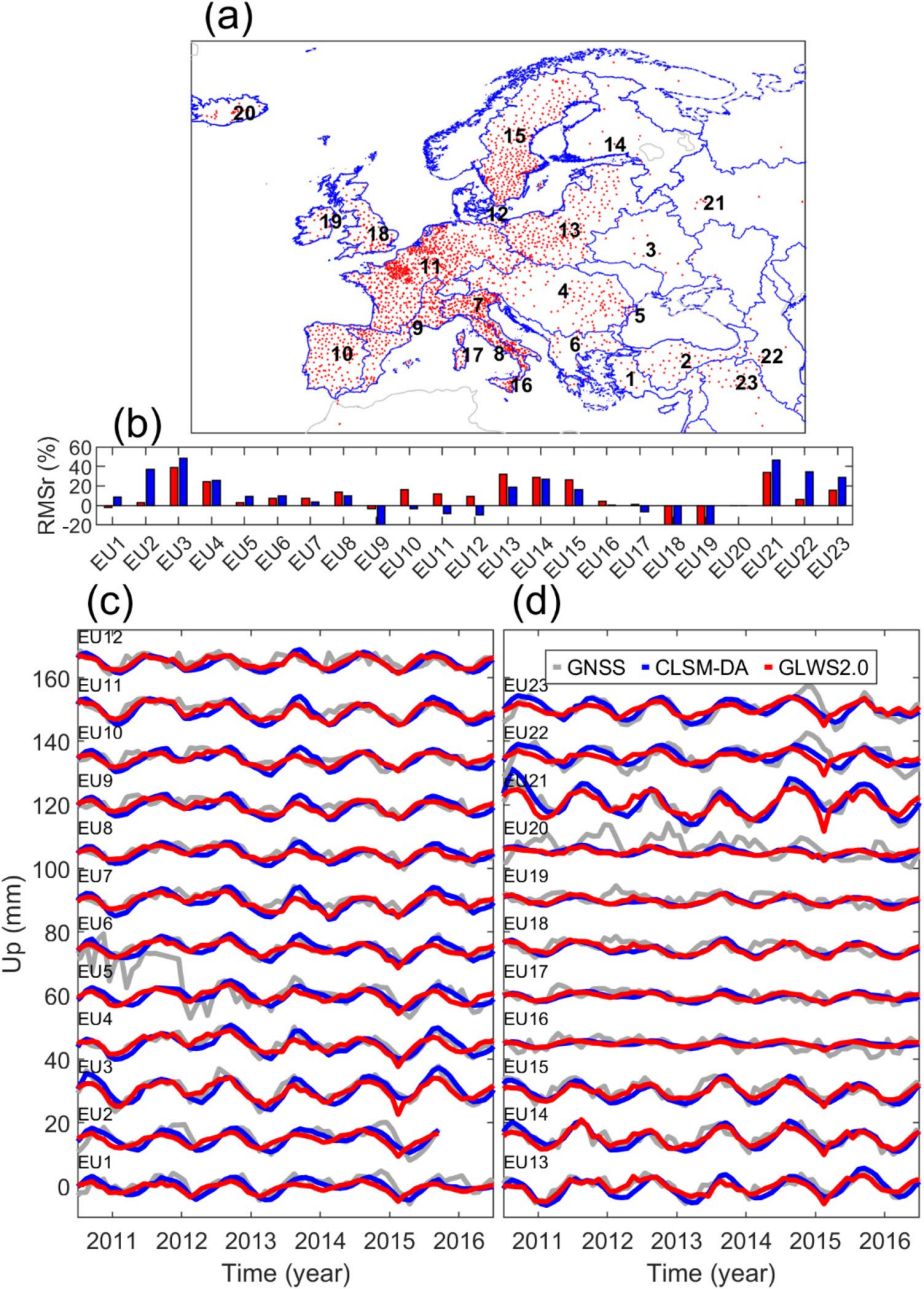
Same as Fig. 6, but for river basins in Europe.

As depicted in Fig. 7b, the loading displacement computed by CLSM-DA better recovers GNSS data in basins located in Eastern Europe. For these basins, the displacement time series computed by the CLSM-DA indicates a more accurate time of maximum annual subsidence, like our findings in western parts of North America. In contrast, there are six basins (i.e., EU9 to EU15) in which the GNSS data is better represented by the GLWS2.0 loading model. For these basins, the smaller seasonal water variation proposed by GLWS2.0 is more consistent with GNSS observations.

For the remaining basins around Italy, UK and Iceland, we do not find a meaningful difference between the agreement of the two GA models and GNSS data, although both models have large uncertainties in Iceland (EU20), as shown in Fig. 7d. Furthermore, GNSS data shows an unusually large subsidence in basins EU1, EU2, EU22 and EU23 around April 2015, as a lagged consequence of the extra heavy rainfall loading in Turkey in November 2014. However, this extra stored water is not accurately captured by the GA models.

##### South America

**Fig. 8:**
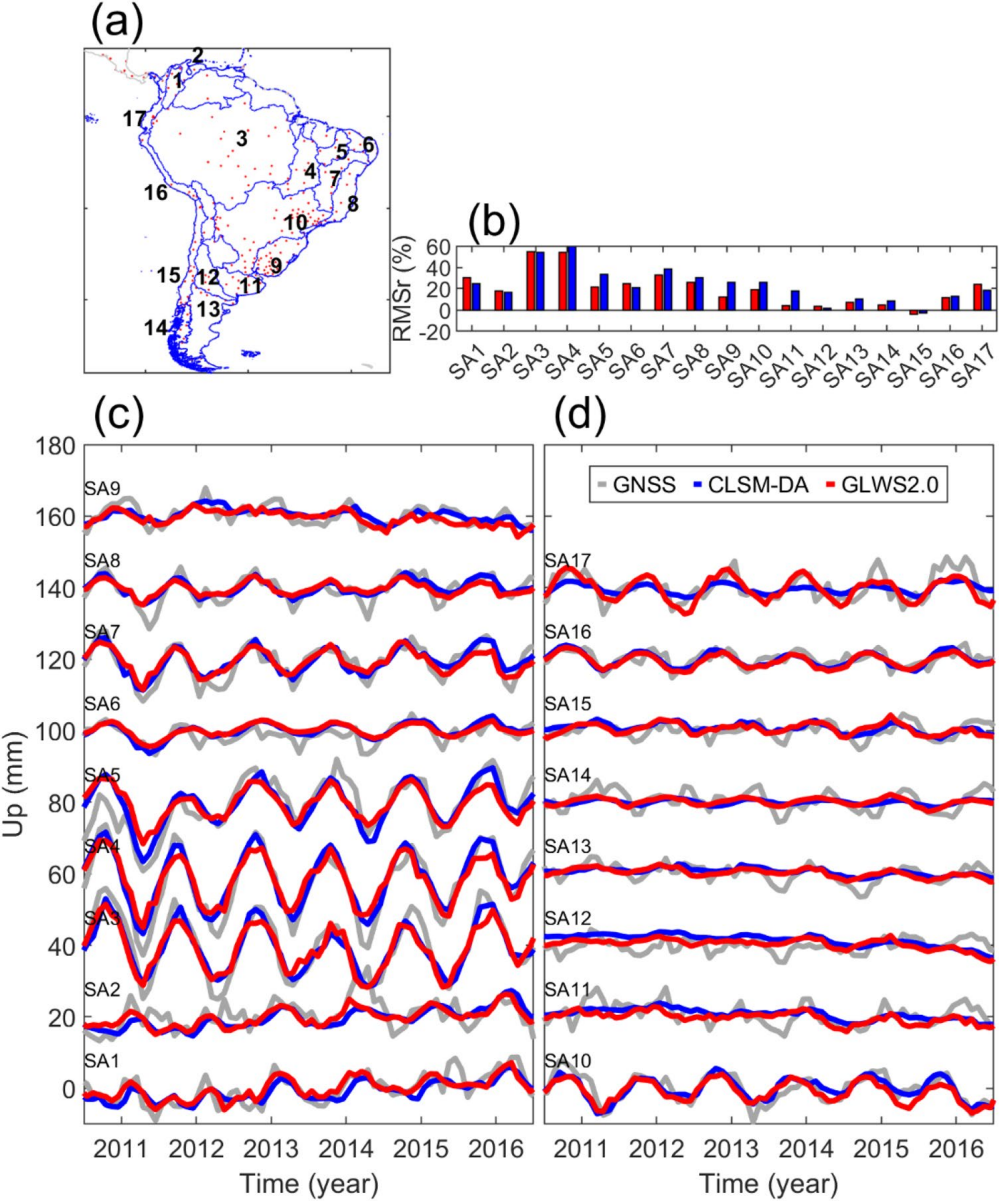
Same as Fig. 6, but for river basins in South America.

Figure 8 demonstrates that the predicted displacement closely resembles GNSS data across basins with strong seasonal water variation, i.e. SA3 to SA5. In SA3, although the GLWS2.0 loading correction causes slightly larger RMSr of GNSS data, Fig. 4 shows a non-uniform model performance variation in this basin. As demonstrated in^54^, the land oscillation at stations near the main tributaries of the Amazon River (in SA3), is primarily induced by the water variation of the river system. Hence, we think that the inclusion of the water bodies in GLWS2.0 has improved its performance at those sites. We also observe a better performance for GLWS2.0 in SA1 and SA17 due to the larger values of water variation proposed by the model.

In contrast, the larger seasonal water variation proposed by CLSM-DA has increased its agreement with GNSS data in SA4, SA5, SA7, SA8, and southeast of SA3 as well as northeast of SA10 (as shown in Fig. 4). In the southern part of South America (i.e., SA9, SA11 to SA14, and southern part of SA15), the annual water variation is small, and the two models’ loading corrections reduce the RMS of GNSS data only marginally. This again highlights the limitations of GA models in capturing local water variations with sub-seasonal frequency.

##### Africa, Asia and Australia

**Fig. 9:**
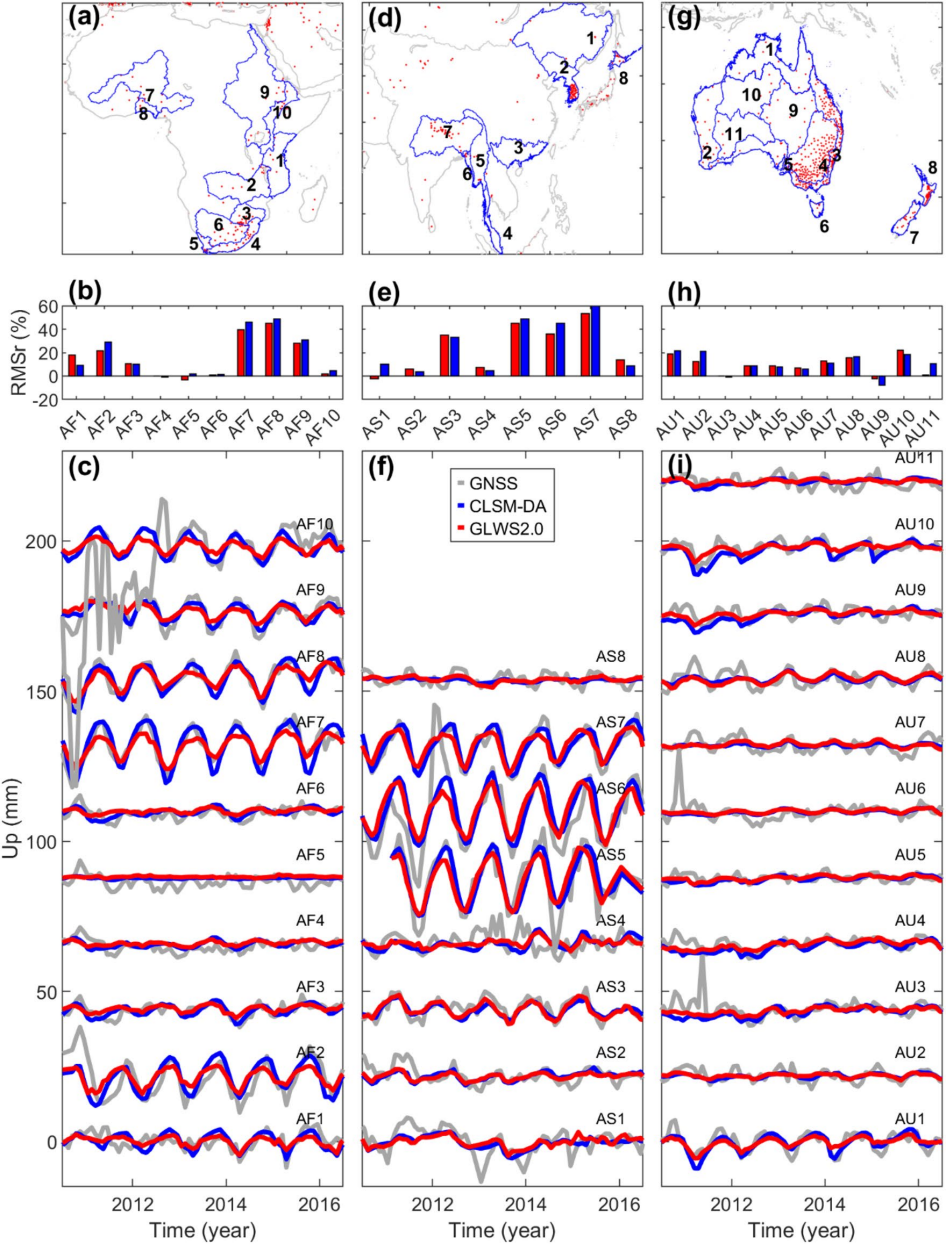
Same as Fig. 6, but for river basins in Africa (left), Asia (middle), and Australia (right).

In this section, we present the results of the hydrological model assessment for Africa, Asia, and Australia, as shown in the left, middle, and right columns of Fig. 9, respectively.

Starting with the African river basins, Fig. 9c shows that the predicted loading displacement closely resembles GNSS observations in regions with substantial terrestrial water storage variations, namely AF2, AF7, AF8, AF9, and AF10. This agreement is particularly notable in AF7 to AF9, but it is degraded in AF10 due to unresolved GNSS offsets prior to 2013.

Across these basins, the predicted amplitude from GLWS2.0 reaches only ~ 52–66% of that from CLSM-DA, with the latter providing better agreement with GNSS-derived displacements. Anomalous uplift is observed in AF2 during 2011 when the CMC calculation includes data from two GNSS stations in Zambia (MONG and TEZI) and one in Malawi (MONG). Our visual inspection confirms the uplift signal in these three sites. In basin AF1, long episodic GNSS data gaps hinder a reliable comparison between models and observations.

In contrast to the abovementioned basins with distinct TWS variation, the drier regions of Southern Africa, particularly AF3 to AF6, exhibit relatively small hydrological loading signals, with GNSS-measured displacements generally below 10 mm peak-to-peak. In these areas, both CLSM-DA and GLWS2.0 show no discernible advantage in recovering GNSS-observed signals.

For the model assessment of the Asian river basins, we exclude AS1, as four out of its five GNSS stations contain long episodic data gaps. In AS2, we also discard seven out of 36 GNSS stations, namely ULLE, DOKD, GEOM, JEJU, MA04, GAG1, and HGDO, as they are located on islands, and thus, may not accurately reflect the hydrological loading signals over the mainland. Furthermore, only two stations in AS2 have GNSS records predating December 2013, which limits the reliability of model assessments during that period.

Despite these limitations, we further analyzed AS2 to understand the extent of agreement between modeled and observed signals. As shown in Fig. 9f, the GA model predictions only weakly follow the GNSS displacement signals in this basin. To better understand this inconsistency, we examined the spatial variability of the displacement time series from all GNSS stations in AS2, and found that several stations such as NONS, KUNW, DON1, YECH, GOCH, and CHC1, exhibited ~ 20–35 mm of GNSS-derived annual vertical land motion between 2015 and 2017. However, the two models predicted only ~ 5 mm of annual displacement at these sites.

Closer inspection of the GNSS time series (Figure A4 of the supplementary material) indicates that (i) the unusually large seasonal oscillations abruptly diminished after a particular date, and (ii) nearby stations located within few tens of kilometers show smaller seasonal variations without such amplitude changes. These two observations suggest that the amplified seasonal signals at the problematic sites are unlikely to reflect real hydrological loading but are instead associated with instrumental or processing effects. A more detailed investigation of the exact cause would require access to station-level metadata and processing logs, which is beyond the scope of this study.

In contrast, other basins such as AS5 (Irrawaddy), AS6 (Indo-Myanmar watershed), and AS7 (Brahmaputra-Ganges) exhibit some of the largest annual water variations globally, as evident from both the modeled and GNSS-observed loading signals in Fig. 9f. In these basins, the CLSM-DA model appears to capture the larger seasonal amplitudes more realistically, aligning with our earlier observations in African river basins.

We assess the performance of the two GA models against GNSS observations in 11 Australian river basins. As shown in Fig. 9i, among these, AU1 stands out as the only basin showing a clear seasonal hydrological loading signal. In AU1, the difference in RMSr of the GNSS-load-corrected time series is less than 5% between the two models. However, the peak-to-peak annual displacement predicted by CLSM-DA aligns more closely with the GNSS-derived signal.

Figure 9i also shows that the land subsidence of the extra water loading from the January 2011 heavy rainfall in northern Australia is detectable by GNSS in AU1, AU9, and AU10. In these basins, CLSM-DA slightly better captures the associated loading anomaly than GLWS2.0, but it tends to generally overpredict the displacement amplitude compared to GNSS observations. This overestimation could arise from aliasing of high-frequency and sub-seasonal signals into the assimilation process.

## Discussion and conclusions

The mean annual amplitude of TWS predicted by GLWS2.0 and CLSM-DA from 2004 to 2019 differs by more than 25 mm across 40% of the global grid cells. Additionally, the timing of maximum or minimum annual storage diverges by over 30 days in half of the model domain. These inter-model inconsistencies in water storage estimates propagate directly into the forward modeling of load-induced surface displacements.

**Fig. 10:**
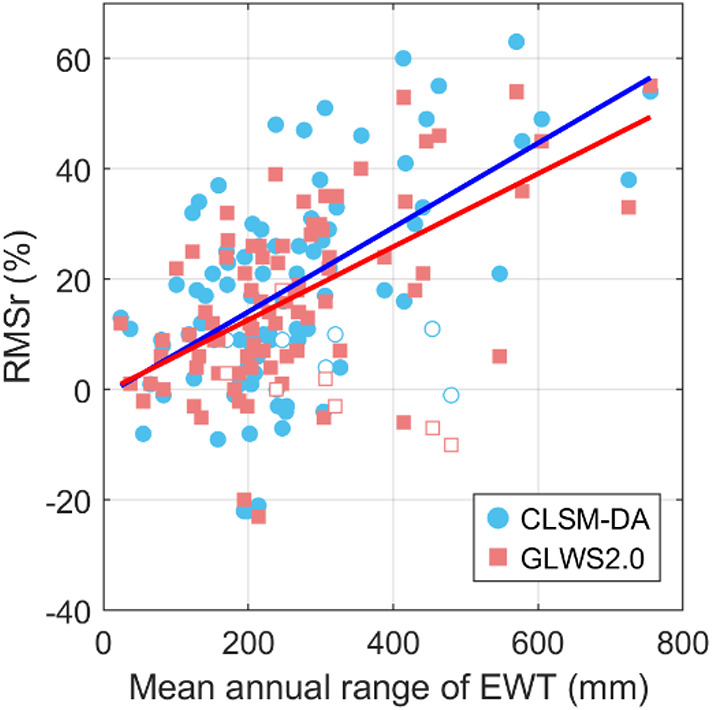
Relationship between water variability and model-GNSS agreement. Open markers represent basins excluded from the regression due to known GNSS data issues. The positive trend of the best-fit lines indicates improved model performance in basins with greater water storage variability, with CLSM-DA demonstrating an overall stronger agreement with GNSS observations.

To quantify model performance, the modeled hydrological loading signal was subtracted from that observed by GNSS, and the RMSr of the load-corrected GNSS data is computed. Additionally, GNSS stations were grouped by river basin, and the RMSr of the median basin-wide displacements was computed, allowing for a regionally focused evaluation. Figure 10 presents a positive correlation between the basin-level RMSr and the mean annual range of EWT. Basins with problematic GNSS data, such as NA15, NA17, EU20, AF1, AF10, AS1, and Iceland, are denoted with open markers and excluded from the regression analysis.

Overall, the correction for modeled hydrological loading reduced the RMS of GNSS vertical displacement across most river basins. However, global hydrological models rely on regionally- averaged meteorological forcings and simplified parametrization of terrestrial water-cycle processes, hindering their ability to capture localized water variations. These limitations are reflected in the RMSr values, which are mostly less than 50%, and in model-GNSS correlations at sub-seasonal timescales, falling below 0.50 in approximately 90% of the analyzed basins. Advances in next-generation Earth observation systems, such as the Surface Water and Ocean Topography (SWOT) mission, are expected to help address some of these limitations^55^.

To better understand the relative strengths of the two GA models, we compared their performance across 76 basins with annual EWT variations exceeding ~ 100 mm (excluding seven basins with poor GNSS data). In this subset, the models performed comparably in 41 basins, with differences in RMSr below 5%. CLSM-DA outperformed GLWS2.0 in 25 basins, while GLWS2.0 yielded better fits to GNSS data in 10 basins. For regions with relatively small annual water variability, no significant difference in performance was observed.

Regionally, GLWS2.0 tends to underestimate annual water variability in major African and Southeast Asian basins. It also exhibits a ~ 30–60 day lead in the seasonal timing of water storage in regions such as the mountainous Western United States and Eastern Europe compared to CLSM-DA and GNSS data. In contrast, GLWS2.0 generally performs better in regions like the Eastern United States and Western Europe, where hydrological loading exhibits a greater sub-seasonal component. In these areas, CLSM-DA often overestimates the annual TWS signal.

The observed differences between the two GA models are a combined effect of three main sources. First, the CLSM-F2.5 and WGHM2.2e models used in the two GA systems differ in model parameterization and climate forcing. Second, unlike the mascon solution used in CLSM-DA, GLWS2 assimilates spherical-harmonic GRACE data that require spatial filtering to suppress correlated errors. This filtering, however, can also attenuate portions of the true hydrological signal. Third, differences in the weighting of GRACE observations within each assimilation framework also influence how strongly the systems adjust toward satellite-derived mass variations.

We suspect that the closer agreement of CLSM-DA with GNSS-derived vertical displacement in hydrologically dynamic regions such as Southeast Asia and Africa is likely influenced by the characteristics and weighting of the GRACE observations in the assimilation system. While this can improve broad-scale TWS representation, it may also broaden the effective spatial footprint of assimilated signals, contributing to the overestimation of TWS anomalies in hydrologically complex regions such as Europe. These results highlight the importance of GRACE preprocessing choices, observation-weighting schemes, and adaptive assimilation methods to preserve local hydrological signals while fully leveraging GRACE’s large-scale information.

This study demonstrates the utility of GNSS-derived vertical displacement data as an independent observational constraint for evaluating global hydrological models. In particular, GNSS provides valuable insights in regions with significant TWS variability, complementing satellite gravimetry. The GNSS records also captured displacement signals associated with episodic heavy rainfall events, signals that were typically underestimated by both models. As GNSS networks continue to expand and multi-GNSS datasets become more widely available, geodetic observations are poised to play an increasingly vital role in hydrological model validation and improvement.

While the inversion of GNSS vertical displacements to estimate TWS remains challenging, especially in regions with sparse station coverage, incorporating these GNSS-inverted TWS anomalies into GRACE-based assimilation frameworks holds promise for overcoming some of GRACE’s limitations. This represents a promising avenue for future research, though optimal assimilation strategies require further investigation.

## Supplementary Information

Below is the link to the electronic supplementary material.

Supplementary Material 1

## Data Availability

The GNSS displacement observations are available in https://geodesy.unr.edu/NGLStationPages/GlobalStationList and ftp://igs-rf.ign.fr/pub/repro3. Loading Green’s functions are provided in https://www.sciencedirect.com/science/article/pii/S0098300412002245#s0065. The GLWS2.0 model is available in https://doi.pangaea.de/10.1594/PANGAEA.954742 and CLSM-DA model can be obtained from https://disc.gsfc.nasa.gov/datasets/GLDAS_CLSM025_DA1_D_2.2/summary. The non-tidal ocean and atmospheric loading data products are available in the ESMGFZ data product repository at http://rz-vm115.gfz.de:8080/repository.
