# Supplementary material for: GNSS evaluation of GRACE-assimilated water storage models over 89 river basins worldwide
Source: Sci Rep. 2026 Jan 29;16:4307. doi: 10.1038/s41598-025-31887-1 (PMC12859144; doi:10.1038/s41598-025-31887-1)
Supplement: Supplementary file 1 — Supplementary Material 1 [file 41598_2025_31887_MOESM1_ESM.docx]

Supplementary Material for ‘GNSS Evaluation of GRACE-Assimilated Water Storage Models Over 89 River Basins Worldwide’


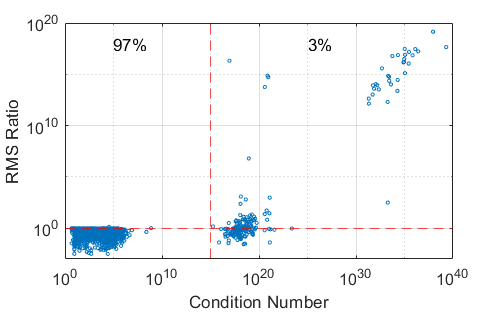


***Fig. A1****: Ratio of RMS of detrended GNSS-derived displacement time series after and before offset correction as a function of condition number which is derived from a Least-Squares regression using the functional model shown in Equation 1. The plot shows a sudden increase in the RMS ratio when condition number exceeds* ${10}^{15}$*( the vertical red line).*


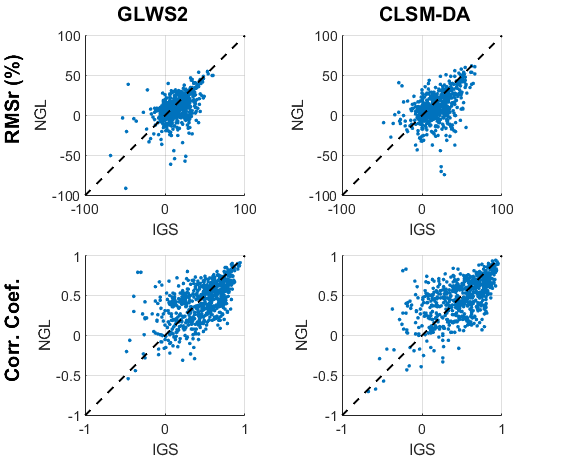


***Fig. A2****: Comparison of model–GNSS agreement metrics derived from IGS and NGL GNSS displacement products at common stations. Each point represents a station’s RMS reduction (top row) and correlation coefficient (bottom row) between GNSS and model-predicted vertical displacements for GLWS2 (left) and CLSM-DA (right).*

*The close clustering around the 1:1 line indicates that the slightly higher noise level in the NGL product does not introduce a systematic bias in model performance evaluation.*

*
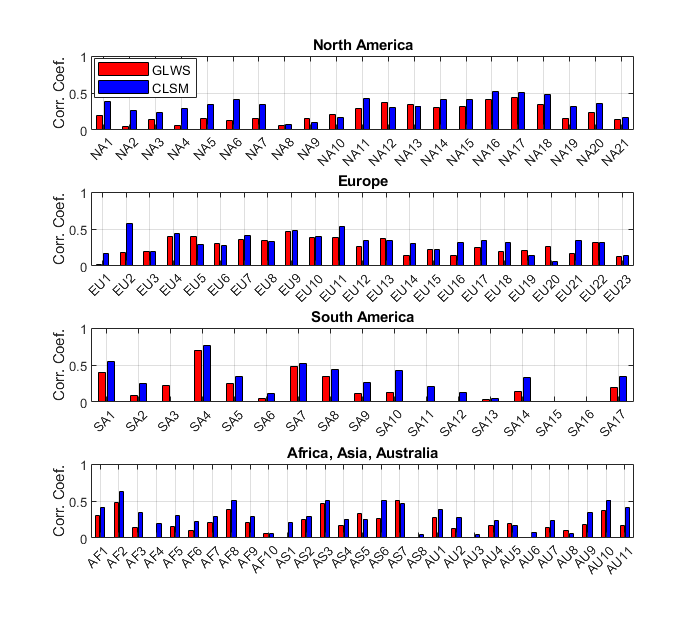
*

***Fig. A3****: Correlation coefficient between modeled and GNSS-derived sub-seasonal hydrological loading displacement computed by wavelet analysis of the displacement common mode component per river basin.*


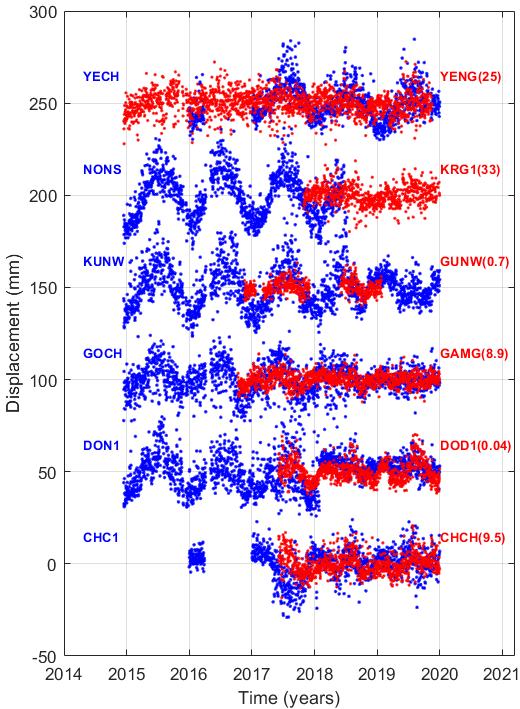


***Figure A4:*** *Comparison of GNSS-derived vertical land displacement time series at several stations in South Korea with unusually large seasonal fluctuations (blue), and their nearby stations (red). The distance between each pair of adjacent stations (in km) is shown in parentheses. The large-amplitude seasonal signals observed at the early part of the blue time series diminish abruptly after a particular date, suggesting that these oscillations are not due to hydrological loading but likely arise from instrumental or processing effects. This interpretation is also confirmed by the smaller seasonal variations at the nearby sites.*
